# Specialist wait time reporting using family physicians’ electronic medical record data: a mixed method study of feasibility and clinical utility

**DOI:** 10.1186/s12875-022-01679-x

**Published:** 2022-04-07

**Authors:** Michelle S. Naimer, Babak Aliarzadeh, Chaim M. Bell, Noah Ivers, Liisa Jaakkimainen, Warren McIsaac, Christopher Meaney, Rahim Moineddin, Joanne A. Permaul, Tutsirai Makuwaza, Sahana Kukan

**Affiliations:** 1 Ray D. Wolfe Department of Family Medicine, Sinai Health, 60 Murray Street, Box 25, Toronto, ON M5T 3L9 Canada; 2 Department of Family and Community Medicine, University of Toronto, Toronto, ON Canada; 3 Department of Medicine, Sinai Health, Toronto, ON Canada; 4 Department of Medicine, University of Toronto, Toronto, ON Canada; 5 Women’s College Hospital, Toronto, ON Canada; 6 Department of Family and Community Medicine, Sunnybrook Health Sciences Centre, Toronto, Canada

**Keywords:** Wait time, Specialist referrals, Primary care

## Abstract

**Background:**

More than 50% of Canadian adult patients wait longer than four weeks to see a specialist after referral from primary care. Access to accurate wait time information may help primary care physicians choose the timeliest specialist to address a patient’s specific needs. We conducted a mixed-methods study to assess if primary to specialist care wait times can be extracted from electronic medical records (EMR), analyzed the wait time information, and used focus groups and interviews to assess the potential clinical utility of the wait time information.

**Methods:**

Two family practices were recruited to examine primary care physician to specialist wait times between January 2016 and December 2017, using EMR data. The primary outcome was the median wait time from physician referral to specialist appointment for each specialty service. Secondary outcomes included the physician and patient characteristics associated with wait times as well as qualitative analyses of physician interviews about the resulting wait time reports.

**Results:**

Wait time data can be extracted from the primary care EMR and converted to a report format for family physicians and specialists to review. After data cleaning, there were 7141 referrals included from 4967 unique patients. The 5 most common specialties referred to were Dermatology, Gastroenterology, Ear Nose and Throat, Obstetrics and Gynecology and Urology. Half of the patients were seen by a specialist within 42 days, 75% seen within 80 days and all patients within 760 days. There were significant differences in wait times by specialty, for younger patients, and those with urgently labelled medical situations. Overall, wait time reports were perceived by clinicians to be important since they could help family physicians decide how to triage referrals and might lead to system improvements.

**Conclusions:**

Wait time information from primary to specialist care can aid in decision-making around specialist referrals, identify bottlenecks, and help with system planning. This mixed method study is a starting point to review the importance of providing wait time data for both family physicians, specialists and local health systems. Future work can be directed towards developing wait time reporting functionality and evaluating if wait time information will help increase system efficiency and/or improve provider and patient satisfaction.

**Supplementary Information:**

The online version contains supplementary material available at 10.1186/s12875-022-01679-x.

## Introduction

Canada has the longest wait times to see a specialist physician according to a recent Commonwealth Fund survey of eleven countries [1]. More than 50% of adult patients wait longer than four weeks to see a specialist, compared to the international average of 36% [1]. Median wait times to see specialists in Ontario range from 39 to 76 days for medical specialists and 33 to 66 days for surgical specialists [2]. Wait times from primary to specialty care in Canada have been increasing and are 155% longer than in 1993 [3]. Prolonged waiting for specialists may be associated with adverse consequences such as increased pain and suffering, inferior medical outcomes, and economic costs from lost wages and productivity [4, 5].

In 2007, Willcox et al. conducted a cross-national comparison of strategies used by countries to measure and reduce wait times [6]. Five countries used supply-side strategies, such as targeting funding toward increased hospital capacity and staff. Some countries used more complex initiatives that addressed demand side techniques, such as using explicit criteria to prioritize access to surgery. The authors recommended that policymakers consider extending the measurement of waiting times to include the point of referral to treatment in order to reflect patients’ actual experience. To date, there are few benchmarks in place targeting wait times from primary to specialty care [5, 7]. In addition, there are limited interventions found in the literature that have shown impact on reducing wait times from primary care to specialty care. Jaakkimainen et al. published a study that outlines patient and provider characteristics of wait times from primary to specialty care [2]. They found that patient factors and most physician factors do not seem to be consistently associated with wait times, except for family physician practice location and practice size.

In Canada, most referrals are directed to specific specialist physicians by primary care providers. In some cases, referrals are made to specialist clinics and the specialist is then selected for the referral by the specialty clinic. Specialist visits are covered by Canada’s universal health insurance plan, and primary care providers can refer patients to any specialist of their choosing. Primary care providers typically select the specialist, and fax or email a referral letter to the specialist’s office using a paper-based system or an electronic health record of their choosing. Historically, there has been little information available to primary care physicians about which specialists have long waits or are available in different regions. Physicians rely on an ad hoc system based on personal experience, word of mouth, electronic medical record (EMR) or internet searches to identify specialists. At times, physicians may refer a patient and find out months later the referral was never received or additional information is needed [8]. Neimanis et al. analyzed 770 referrals and found that 36.4% of referrals received no response within a 5 to 7 week period [8]. Access to accurate wait time information may help family physicians choose the most suitable specialist to address a patient’s specific needs.

### Objective

This mixed methods study assessed the feasibility of creating primary to specialist care wait reports from primary care electronic medical records (EMRs). The primary outcome was the median wait time from physician referral to specialist appointment for each specialty service. The clinical utility of the resulting wait time data was evaluated through focus groups with family physicians and specialist physicians [9].

## Methods

### Study setting

A convenience sample of two primary care clinics from the University of Toronto Practice-based Research Network (UTOPIAN) [10] were recruited for this study: an academic family health team in downtown Toronto, Ontario (caring for approximately 12,000 patients of all ages), and a community primary care clinic in Vaughan, Ontario (caring for approximately 10,000 patients of all ages). Both are affiliated with Sinai Health and utilized the same EMR (Nightingale), at the time of the study. We included both sites to study community and downtown academic family practice perspectives and to include data from diverse referral networks. Ethics approval was obtained from the Sinai Health Research Ethics Board.

### Data collection

Patients from the two study sites were included if they had one or more referral records between January 1^st^, 2016 and December 31^st^, 2017, and if they had at least 2 visits in 3 years prior to December 31^st^, 2017. Only patients who were not deceased by the end of the study period were included. Age was calculated at January 1^st^ 2016. The unit of analysis is a “visit”, where multiple visits can be nested for a patient. For example, one patient might have referrals to both cardiology and endocrinology, possibly over different visits during the study window. The data set was created by entering the back end of the electronic health record and extracting fields from patient referral. Variables in the final dataset included: family practice location, age, sex, urgency of visit coded to 4 categories (as per availability, within 1 month, within 1 week and urgent), specialist name, specialty, specialist department or clinic, specialist address, referral date, referral status, patient postal code and appointment date. Wait times were defined as the number of days between referral date and appointment date. Routine referrals were considered those indicated by the referring physician “as per availability” and “within 1 month”. Urgent referrals included those marked “urgent” and “within 1 week”. Referral data was further cleaned by manual review by the lead study author (MN) to remove referrals with an unknown site, missing department/speciality or to a non-medical or surgical specialty (for example, referrals to physiotherapy or optometry) and referrals where the wait time was missing. Where referrals to a specific specialty were fewer than 50 patients, specialities were grouped into an “other” category. Wait times were calculated by taking the appointment date and subtracting the number of days from the referral date. To validate the accuracy and completeness of the wait times calculated, a random sample of 150 referrals was selected and a research assistant reviewed the charts to assess the agreement between wait times calculated through EMR data that was extracted, cleaned and coded, and wait times calculated through manual chart review (the gold standard).

### Creation of wait time reports

The format of the wait time report was developed with input from the study investigators. A sample report is included in Appendix 1 and has been anonymized for purposes of publication.

### Statistical analysis

#### Quantitative analysis

Wait times did not have a normal distribution therefore the analysis included the calculation of median wait times and inter-quartile ranges [2]. Median wait times were calculated and stratified by specialist/department and by practice location. Exploratory, bivariate analyses were conducted examining the median wait time including completeness of wait time data and patient or physician characteristics that may have contributed to wait times such as patient age or sex, patient income level before taxes [11], combined material and social deprivation index [12] or physician age, sex and location of practice. Area-level indices such as income quintiles, and the material and social deprivation index were derived using patients’ 6-digit postal codes. Non-parametric Wilcoxon rank-sum tests and Kruskal Wallis tests were used to assess the association between these patient/provider characteristics and median wait-times. We calculated the proportion of referrals that were seen within a benchmark of 18 weeks (a benchmark used in the United Kingdom where 90–95% of referrals are targeted to be booked within 18 weeks) [13]. All statistical analyses were conducted at an alpha of 0.05, using SAS version 9.4.

#### Qualitative analysis

A qualitative approach informed by grounded theory was used [14]. A review of the literature helped to inform the design of the qualitative aspect of the study [15, 16]. Focus groups were used to capture the subjective meaning and utility of the wait time reports that were created. Specialists from the top 7 specialties referred to by family physicians at both practice sites were invited to participate in a focus group. The aim was to have 6–8 participants in the specialist focus group, and in each of the Toronto and Vaughan family physician focus groups. Individual interviews were scheduled with participants who were unable to attend a focus group. Focus groups and interviews were conducted in-person by the qualitative research assistant and the Principal Investigator. At the start of the session, physicians received a paper wait time report to review that included median wait times of specialists (anonymized) by practice address for 7 specialty areas. Specialists received a similar paper report plus a more detailed confidential report with their personal wait times listed for each referral made to them specifically. A semi-structured interview guide was used for family physicians (Appendix 2) and specialists (Appendix 3) that asked about the relevance, clinical utility and acceptability of the wait time reports. Focus groups and interviews were conducted over 2 months from October to November, 2018. Interviews lasted 60 min and were audio-recorded and transcribed verbatim, anonymized and analyzed using techniques informed by grounded theory [14] including coding, interpretations of data patterns and constant comparison method [17].

Research team members performed line by line open coding of interview transcripts [18] using an inductive approach with principles of constant comparison [19] for analyzing data. Co-investigators (M.N. and T.M.) read the same transcripts independently and met with one another to develop a coding manual. The transcripts were subsequently coded line by line by the qualitative researcher (T.M.). Team members met to review and refine codes and any discrepancies were resolved through discussion. Inductive analysis was appropriate for use in this research since there were no previous studies dealing with the phenomenon under investigation [20]. NVivo 11 (NVivo qualitative data analysis Software; QSR International Pty Ltd. Version 11, 2016) was used for data management.

## Results

### Quantitative analysis

**Fig. 1:**
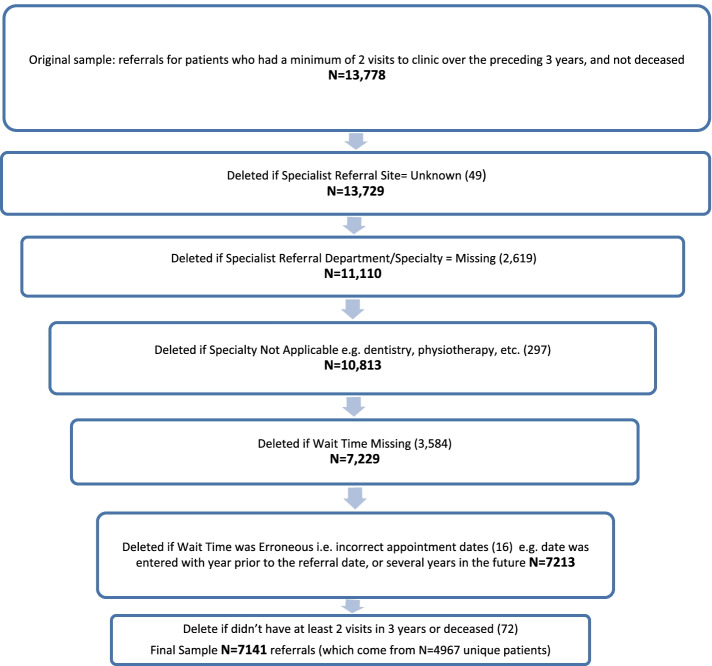
Wait time data extraction process

Figure 1 summarizes the wait time data extraction and cleaning process from the EMR. The data cleaning process required some manual review of spreadsheets to remove some referrals as noted in the Figure. It took approximately 2–3 h of time to identify and remove missing referrals, and specialties that were not applicable. Validation of wait time calculations from 150 charts demonstrated a wait time concordance rate of 100%. Referrals analyzed included referrals where appointments dates were pending or not recorded in the EMR. The final sample of specialist referrals where wait time information was available included 7141 referrals (4967 unique patients).

Table 1 outlines characteristics of the referrals. Wait times differed across age groups. We observed differences in median wait times in those > 65 years (46.0 days) versus those 0–19 years (40.0 days) (*p* = 0.0006). We also observed differences in median wait times in those > 65 years (46.0 days) versus those 20–44 years (42.0 days) (*p* = 0.0002). There was no difference in wait times by sex. Wait times for referrals marked as urgent had a shorter wait time compared to routine referrals: 13 days versus 43 days respectively (*p* < 0.01). There seems to be some cyclicality to wait times based on season referred, with a longer wait in the summer season, however, more robust data using more referrals and practice sites would be needed to examine this trend further. Analysis of income level and material and social deprivation level for patients with an available postal code did not show any meaningful difference in wait times by income quintile before taxes, or by material and social deprivation.

**Table 1:** Comparison of wait times by patient and referral characteristics (*N* = 7141)

**Characteristic**	**N**^**b**^** (%)**	**Minimum**	**25th Pctl**^**c**^	**Median**^**d**^	**75th Pctl**^**e**^	**Maximum**^**f**^	***P***^**h**^
Referral Site:							
• Mount Sinai Downtown Academic FHT^a^	4242 (59.4)	1.0	21.0	42.0	78.0	760.0	< 0.01
• Sherman Health and Wellness Community FHT	2899 (40.6)	1.0	25.0	43.0	83.0	561.0	
Sex:							
• Female	4501 (63.0)	1.0	22.0	43.0	79.0	760.0	0.34
• Male	2640 (37.0)	1.0	21.0	41.0	83.0	616.0	
Age group of patient referrals (years)							
• 0–19	410 (5.7)	1.0	25.0	46.0	84.0	400.0	< 0.01
• 20–44	2012 (28.2)	1.0	24.0	43.0	83.0	561.0	
• 45–64	2428 (34.0)	1.0	22.0	42.0	81.0	760.0	
• 65 +	2291 (32.1)	1.0	20.0	40.0	77.0	616.0	
Urgency of Referral^g^							
• Routine	4471 (62.6)	1.0	23.0	43.0	81.0	616.0	< 0.01
• Urgent	296 (4.1)	1.0	6.0	13.0	29.5	469.0	
• Urgency Missing on Referral	2374 (33.2)	-	-	-	-	-	
Season/Year of referral							
• Winter 2016	760	1.0	20.5	38.0	77.0	746.0	< 0.01
• Spring 2016	943	1.0	21.0	41.0	84.0	441.0	
• Summer 2016	802	1.0	25.0	47.5	86.0	760.0	
• Fall 2016	789	1.0	21.0	45.0	82.0	454.0	
• Winter 2017	843	1.0	19.0	36.0	71.0	407.0	
• Spring 2017	1055	1.0	22.0	41.0	78.0	451.0	
• Summer 2017	978	1.0	26.0	46.0	82.0	458.0	
• Fall 2017	971	1.0	24.0	44.0	84.0	532.0	
Income quintile before taxes							
• 1 (Lowest)	748 (11.0)	1.0	21.0	41.0	73.5	458.0	0.16
• 2	942 (13.8)	1.0	22.0	43.0	79.0	491.0	
• 3	939 (13.8)	1.0	23.0	45.0	88.0	760.0	
• 4	1815 (26.6)	1.0	22.0	42.0	80.0	746.0	
• 5 (Highest)	2371 (34.8)	1.0	22.0	43.0	83.0	616.0	
Material and social deprivation index							
• 1 (Least)	1401 (26.6)	1.0	22.0	45.0	88.0	561.0	0.06
• 2	1138 (21.6)	1.0	22.0	41.0	77.0	616.0	
• 3	1369 (26.0)	1.0	21.0	42.0	78.0	760.0	
• 4	676 (12.8)	1.0	21.0	42.0	80.0	432.0	
• 5 (Most)	683 (13.0)	1.0	22.0	42.0	78.0	458.0	

^a^*FHT* Family Health Team, ^b^Denominators may differ between referral characteristics due to missing data, ^c^*25*^*th*^* Pctl* 25^th^ percentile, ^d^minimum: minimum wait time, ^e^*75*^*th*^* Pctl* 75^th^ percentile, ^f^*maximum* maximum wait time, ^g^Routine referrals include referrals indicated by the physician to be “as per availability” and “within 1 month, while urgent referrals include those marked “urgent” and “within 1 week”, ^h^*P*-value based on Wilcoxon rank-sum test for variables with 2 levels, or *P*-value based on Kruskal–Wallis test if characteristic has > 2 levels

Of the 4967 unique patients, 69% had one referral, 22% had 2 referrals, 6% had 3 referrals and 3% of patients had 4 or more referrals. There were 1357 unique specialist names in the database and 596 unique departments. The list was reduced to 33 unique specialities by grouping similar specialty services/departments (Table 2). The top 10 specialties consulted were Dermatology, Gastroenterology, Ear Nose and Throat, Obstetrics and Gynecology, Urology, Ophthalmology, Immunology, Orthopedics, General Surgery and Rheumatology.

**Table 2:** List of specialties referred to and their wait time distribution (*N* = 7141)

**Specialty**	**Number of Referrals**	**%**^**a**^	**Minimum**	**25th Pctl**^**b**^	**Median**^**c**^	**75th Pctl**^**d**^	**Maximum**^**e**^
All Specialities	7141	100	1.0	22.0	42.0	80.0	760.0
Dermatology	1405	19.7	1.0	18.0	34.0	63.0	746.0
Gastroenterology	1040	14.6	1.0	21.0	41.0	82.0	616.0
ENT	673	9.4	2.0	21.0	35.0	64.0	561.0
Ob/Gyn	584	8.2	1.0	30.0	52.0	87.0	458.0
Urology	321	4.5	3.0	36.0	75.0	112.0	551.0
Ophthalmology	309	4.33	1.0	18.0	38.0	62.0	451.0
Immunology	288	4.0	3.0	35.0	70.0	111.5	213.0
Orthopedic Surgery	269	3.8	1.0	15.0	37.0	71.0	760.0
General Surgery	241	3.4	1.0	16.0	41.0	71.0	323.0
Rheumatology	221	3.1	1.0	35.0	62.0	99.0	228.0
Neurology	209	2.9	1.0	31.0	51.0	100.0	407.0
Endocrinology	201	2.8	1.0	29.0	54.0	97.0	469.0
Cardiology	182	2.5	1.0	21.0	38.0	79.0	215.0
Plastic Surgery	175	2.5	1.0	37.0	59.0	100.0	259.0
Psychiatry	170	2.4	2.0	22.0	40.5	63.0	400.0
Sports Medicine	129	1.8	2.0	15.0	24.0	37.0	441.0
Hematology	92	1.3	3.0	26.5	56.5	97.5	237.0
Urogynecology	92	1.3	2.0	32.0	51.5	96.0	452.0
Sleep Clinic	87	1.2	2.0	22.0	46.0	88.0	400.0
Nephrology	73	1.0	3.0	16.0	22.0	52.0	388.0
Respirology	71	1.0	8.0	26.0	50.0	77.0	216.0
Vascular Surgery	56	0.6	5.0	23.5	48.0	76.5	161.0
Physiatry	48	0.7	1.0	53.0	70.0	100.5	219.0
Pediatrics	44	0.6	1.0	14.0	32.0	48.0	114.0
Genetics	43	0.6	9.0	58.0	101.0	186.0	532.0
Geriatrics	21	0.3	8.0	24.0	42.0	55.0	399.0
Oncology	21	0.3	8.0	15.0	23.0	52.0	121.0
Internal Medicine	19	0.3	2.0	9.0	19.0	58.0	219.0
Pain Clinic	19	0.3	1.0	40.0	75.0	165.0	546.0
Neurosurgery	17	0.2	14.0	31.0	49.0	104.0	439.0
Hepatology	11	0.2	10.0	32.0	79.0	156.0	159.0
Infectious Disease	9	0.1	18.0	27.0	40.0	47.0	227.0
Palliative Care	1	0.01	21.0	21.0	21.0	21.0	21.0

^a^*%* percentage, ^b^*25*^*th*^* Pctl* 25^th^ percentile, ^c^*minimum* minimum wait time, ^d^*75*^*th*^* Pctl* 75^th^ percentile, ^e^*maximum* maximum wait time

**Fig. 2:**
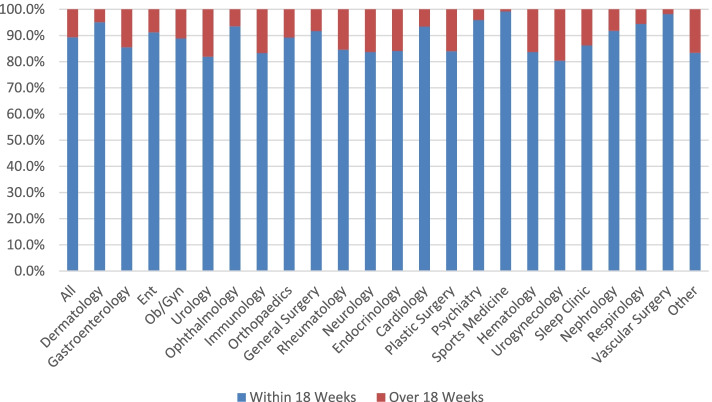
Wait times within 18 weeks and over 18 weeks by specialty

The median wait time of all referrals was 42 days, 75% of patients were seen within 80 days and all patients were seen within 760 days. Almost 90% of patients saw the specialist within an 18 week benchmark, and 99.53% of referrals were seen within the year (Fig. 2).

### Qualitative analysis

We conducted three focus group sessions, two family physician-centred focus groups and one specialist-centered focus group, along with two one-on-one specialist interviews. In total, 6 specialists and 14 family physicians participated in the focus groups. Socio-demographic surveys were completed by 17 of the 20 (85%) participants (Table 3). Thirteen of the 17 respondents (76%) were based at academic type practices, with years in practice varying from 4.5 to 21 years. Table 4 summarizes the information obtained from the focus groups and interviews organized by topic area discussed in the semi-structured interviews.

**Table 3:** Sociodemographic information of focus group participants

	MSH^a^ FPs^b^ N (%)	Vaughan FPs N (%)	MSH Specialists N (%)
# Attended Focus Group	10	4	6
# Completed Questionnaire	8 (80.0%)	4 (100%)	5 (83.3%)
Gender			
Male	2 (25.0%)	1 (25.0%)	5 (100%)
Female	6 (75.0%)	3 (75.0%)	0 (0.0%)
Age (years)			
31–40	2 (25.0%)	4 (100%)	1 (20.0%)
41–50	2 (25.0%)	0 (0.0%)	1 (20.0%)
> 50	4 (50.0%)	0 (0.0%)	3 (60.0%)
# Years in Practice			
Mean	23.8	4.5	14.2
Range	4–38	3–6	4–25
Mean # Years at Current Location	21.1	4.0	14.0
Practice Type			
Academic	7 (87.5%)	1 (25.0%)	5 (100%)
Community	0 (0.0%)	3 (75.0%)	0 (0.0%)
Combined	1 (12.5%)	0 (0.0%)	0 (0.0%)

^a^*MSH* Mount Sinai Hospital, ^b^*FPs* Family Physicians

**Table 4:** Emerging themes from primary care providers and specialists

**THEMES**	**Sub Themes from Primary Care Providers**	**Sub Themes from Specialists**
**GENERAL IMPRESSIONS**	**Wait time data is viewed positively by primary care providers (PCPs**^a^**) and is seen to have value** 1. *Official registry/access to specialist wait time data seen as very useful*	**Specialists have interest in wait time data** 1. *Some clinics/specialists have never seen their own data or that or colleagues* 2. *Website with specialist by region, updated every month by clinics/specialist seen as useful particularly for PCPs*
**CLINICAL UTILITY**	**Majority of primary care providers perceive wait time data to have significant clinical utility** *• Knowledge of wait-times increases PCP (& patient) specialist choices* *•* Choosing specialist within closest geographical convenience for patient *•* Choosing specialist with shortest wait times *•* Helps to manage patient expectations *• Useful for improving patient care* *•* Useful for urgent referrals *•* Potentially increases patients’ timely access to care—patients don’t have to suffer unnecessarily for long periods	**There was variable perception about` the clinical utility of wait time data for specialists** 1*. Review of data may lead to clinical improvements* *•* Provides feedback and could reveal inefficiencies rather than capacity issues which would allow for redistribution of resources for some specialties *•* If specialists are aware of data they may be motivated to find resolutions (competitive response) *•* Would allow specialists to provide alternatives for patients when full *•* No standardization exists to triaging patients, therefore a review of the triage practices of those specialists with shorter wait times could be helpful for all 2. *Review of data has little or no impact/clinical utility for clinics/specialists* *•* Even if aware, specialists may not be able to change wait times (e.g. Surgeons are limited by how may surgeries they can perform per day) *•* Data would not change practice or how patients are booked *•* Some data (e.g. surgical oncology) already exists at system level yet no changes have been effected, thus specialists question impact of additional data
**PERCEIVED BENEFITS & VALUE OF DATA**	**Data is perceived to have value for both PCPs and specialists because it increases awareness** 1. *Data increases PCP access to knowledge & information regarding specialists (SPs)* *•* Some PCPs currently find specialists by informal methods i.e. asking colleagues who they refer to or asking patients who they friends have been referred to – official registry is useful to have *•* Useful resource when new to the city or newer to practice *•* Organized & accessible—some PCPs are currently find specialists via ad hoc means, i.e. asking colleagues who they refer to or patients who they friends have been referred to 2. *Data allows specialists/clinics to become aware of their own wait times* *•* If specialists are aware of data they may find internal resolutions or workarounds *•* Acknowledgement that specialists may not be able to change wait times even with greater awareness **Wait time data has systemic relevance** 1. *Relevance is seen for local, regional and provincial administration bodies (department chiefs, hospital CEOs, standards committees, LHINs*^b^*, HQO*^c^*)* 2. *Provincial wait time data would highlight inequality in Ontario which may lead to improvements*	**Data increases awareness—availability of data allows specialists/clinics to become aware of their own wait times** 1. *Urgent wait times are often of importance to specialists because of targets set by clinic – most specialists don’t know if they are meeting these* 2. *Data may highlight disconnect between perception (self-reported wait times) and reality* 3. *Sub-specialization wait time data provides more detail of where bottlenecks might be* 4. *Data provides a comparator with other specialties in region even if clinics/specialists are limited in resolutions* 5. *Valuable information, particularly for non-surgical specialists (e.g. Ortho surgeons have Wait 1 & 2 Data provincially monitored and sent back—non surgical ortho data may be lacking)* **The systemic relevance of additional wait time data is not clear for all specialists** 1. *Some data (e.g. surgical/oncology) already exists at system level, yet no changes have been effected, thus specialists question the impact of additional data* 2. *Some specialists acknowledge systemic relevance for department chiefs, hospital CEOs, standards committees LHINs and administrative bodies such as HQO* *•* Primarily useful for department chiefs with regards to bottleneck identification
**PERCEIVED CHALLENGES/LIMITATIONS**	**Data does not provide information related to quality of referral and variances in wait times** 1. *Quality or cannot be discerned* 2. *Data provides no contextual information which may account for outliers* **Wait time data may not change referral patterns—some PCPs report having “favourites” who they already refer to and accessing wait time data may be** **There may be unintended consequence of making data available** 1. *Wait times for some specialists could increase if a real-time or a dynamic, updatable interface is not embedded in the design* 2. *Alienation of some specialists, particularly those with longer wait times*	**Data does not reflect variance with regards to referrals** 1. *Factors influencing referrals and wait time data include* *•* Patient preference *•* PCP preference *•* Type of problem or diagnosis *•* Appropriateness/quality of referral
**RECOMMENDATIONS**	**Presentation of wait time data for PCPs can be improved by increasing clarity, accessibility and user friendliness for PCPs** 1. *Increase clarity* *•* Group data for easier or quicker identification e.g. wait times below or above 50 days *•* Cluster by sub-specialty *(*e.g. separate ob & gyn wait times as difference exists) 2. *Sort data by shortest wait times and/or geography* 3. *Provide filters (i.e. if platform is interactive) so PCPs can choose specialist based on own criteria (e.g. geography, affiliation, urgent/non urgent referral *etc.*) which may change case by case* 4. *PCPs prefer data to be made available to them *via* easy to access means* *• Link in the EMR* *• Email* *• Easy access at point of care important* **Wait time data that is updated regularly is seen as important** 1. *Real time interface and updates if possible was seen as ideal* 2. *The most popular interval for receiving updates was quarterly followed by every 6 months* 3. *An opt out option for PCPs who no longer wish to receive updates is important* **Although benchmarks are key for establishing a standard of care, setting these for specialist referrals may be controversial & challenging to achieve** 1. *Benchmarks may be unrealistic* *• May be unrealistic because of geography and location of specialists—may set up unreasonable expectation* *• There are too many specialties & sub-specialties for benchmarks* *• Specialists in Ontario are at capacity so they can’t accommodate patients any faster* 2. *Benchmarks may inherently place judgement, blame & stress on specialists* 3. *Rather than benchmarks, it is better to look at similar healthcare systems doing better and find ways of mirroring or learning from them* **Public reporting of wait times may be inevitable in the future but may cause challenges (e.g. patients wanting to choose a different specialist than recommended) and further strain the health care system** **For next steps, consider an implementation trial exploring actual utility of wait time date by PCPs and also patient behavior**	**Additional, more robust data may be of interest to both specialists and PCPs** 1. *Contextualized comparison data (to help explain anomalies or outliers) is important* 2. *Data should include communication wait times from specialist to PCP (i.e. letter from specialist to PCP) as this is important for adhering to standards* 3. *Including the type of referrals accepted by may be educational for PCPs* **Quarterly updates of accessible wait time data could allow specialists to review and plan** 1. *Nominal reporting should be by group/clinic consensus* 2. *Data should be easily accessible e.g. email or presentation in specialty group meeting* 3. *Platforms with access barriers e.g. passwords would prevent or limit use of information* **Mixed views exist about establishing benchmarks** 1. *While benchmarks are seen by some as arbitrary because they depend on sub-specialty or diagnosis, they are also seen as important for setting standards and improving healthcare systems* **Public availability all wait time data is OK (and some exists already but should include education for patients so the public better understands clinic variances** **Next steps** 1. *Consider a block randomization trial—releasing data to a few to see if this has impact on actual wait time* 2. *Evaluation of what constitutes urgent referrals are needed as PCPs have a low criteria for “urgent”* *•* Important for PCPs to ask the right questions and know when referral is valid—impacts wait times

^a^*PCP* Primary Care Physician, ^b^*LHIN* Local Health Integration Network, ^c^*HQO* Health Quality Ontario

General impressions of wait time reports by family physicians were that the data was viewed positively and seen to have value. Family physicians perceived that having wait time information had significant clinical utility when deciding upon which specialist to refer. Some physicians reported that in non-urgent cases, they would refer to their preferred specialist- this was often based on familiarity or a prior good working relationship. However, referring a patient to a specialist with a shorter wait, particularly when patient quality of life was of concern was seen as important by most, for example if the patient was in pain or had elevated anxiety. The following comment exemplifies this:

*“A non-urgent referral doesn’t mean that it can wait a year to be seen. It just means they don’t have to be seen tomorrow. But if they are having significant sinus symptoms, and I’ve done everything in my arsenal to help them, do they need to be seen tomorrow for sinus symptoms? No. But I also don’t want them to wait 4 months to see somebody and suffer for 4 months needlessly if they could see somebody within 2 weeks. So that would be super helpful information in my opinion.” (Family Physician)*

Wait time information could also provide a means for family physicians to learn of new specialists and manage patient expectations around waiting for the referral. Having easy access to data at the point of care was identified as important to family physicians (versus paper reports or an email with wait time information).

Wait time information was viewed as an important piece of information by both family physicians and specialists for local, regional and provincial planning.

*“I think it’s amazing. It seems like this would be really useful to be out there both for like a* [specialty named] *association, for government, for LHINs* [Local Health Integration Network]. *I’ve never seen data like this. So I think it would be incredibly useful.” (Specialist)*

General impressions of wait time reports by specialists were that seeing the wait time data was interesting. Some specialists had never seen their own wait time data or that of their colleagues. Specialists had variable perceptions about the clinical utility of wait time data. The data could reveal system inefficiencies and allow for redistribution of referrals for some specialties. However, even if aware, specialists acknowledge they may not be able to change their practice or appointment booking processes.

*“When you’re looking at a process, it’s either inefficient or it’s a capacity problem. And at least from the* [specialty named] *side of things, there are some reasonable evidence based on a glance of the distribution that it’s an inefficiency problem. So certain people are holding up the line, and certain people are not. And they’re not being redistributed that way. But that’s the way referrals have been made in Ontario for the last 200 years. And so that’s why I think there’s a lot more push now to create programs like a rapid assessment clinic. A lot of groups now are sort of first-come, first-serve, depending on individual practitioner wait times.[…]So I guess the way I look at it is that it just confirms a lot of people’s suspicion that within this is probably not a capacity issue right now, it’s probably an inefficiency of distribution issue.” (Specialist)*

Focus groups and interviews furthermore highlighted that specialists may be unaware of their own actual wait times and levels of triaging referrals by specialist offices can vary widely. Specialists stated that sub-specialist wait times would be another key piece of data for additional analysis to help determine where system bottlenecks may be. A perceived limitation of the wait time report was that the data does not reflect variance with regards to referrals. For example, factors influencing referrals and wait times include patient preference, primary care provider preference, type of problem or diagnosis and appropriateness of the referral.

*“It could be perceived that Dr. X with a wait time of 190 days is bad. But it might be that, you know, they are the only person who does that. Or b) they have… they’re doing a really good job, and a lot of people want to go to them, and they don’t want to go anywhere else, and they want to wait.”*

Having access to additional, more robust data was of interest to both specialists and family physicians.

There were mixed views about establishing wait time benchmarks. While benchmarks are seen by some specialists as arbitrary because they depend on sub-specialty or diagnosis, they were also seen as important for setting standards and improving healthcare system. Family physicians thought benchmarks may be unrealistic because of geography and location of specialists, and may set up unreasonable expectations. Benchmarks may also inherently place judgement, blame and stress on specialists. It may be better to look at similar healthcare systems doing better and find ways of mirroring or learning from them.

## Discussion

We conducted a mixed-methods study to evaluate the feasibility of producing primary to specialist care wait time reports from the EMR and conducted focus groups and interviews to determine the clinical utility of the wait time information. Primary care EMRs house valuable specialist wait time data, which has been an untapped resource in most jurisdictions. Interviews with family physicians and specialists demonstrated the relevance and clinical utility of wait-time related information, especially for family physicians.

The 2 year wait time data extracted from the health records of patients in both clinics closely resembles broader trends in specialist referral patterns in the Province. Our top 10 list of specialist referrals varied slightly from another Ontario study published in 2017 that examined specialist referrals in Ontario [21]. Our list includes rheumatology in the top 10, whereas the other study includes cardiology and plastic surgery. Our top 10 list of specialties was used to form the list of specialists that were invited to the specialist physicians focus groups/interviews. On further evaluation of our wait time data, there was no meaningful difference detected in wait times when comparing low income to high income populations, or by least material and social deprivation to greatest material and social deprivation level. There was no association between patient sex and referral wait times, however there was an association between patient age and wait times, with older patients having shorter wait times compared to younger patients. A possible difference was detected by season referred, with summer months resulting in longer wait times, although more robust data involving more years and referrals would be needed to definitively conclude these trends. These results are similar to information found in a 2014 study examining specialist wait times in Ontario. Although slightly different measures of socioeconomic status were used, specialist wait times in this study did not vary by patient income or social and material deprivation scales [2]. It was reassuring to note that wait times were not impacted by a patient’s income level in Canada’s single payer system. In terms of the wait times themselves, the overall median wait time in this study was 42 days and the 75^th^ percentile was 82 days. Our range is 34–75 days for the median wait time compared to the median range 33–76 days in 2014, and 75^th^percentile of 62 to 112 days compared to 63 to 231.5 days in the 2014 study [2]. Interestingly, triaging does seem to happen on a system level with a median wait time that is 30 days less for urgent versus routine appointments. With approximately 94% of referrals being marked as “routine”, family physicians in these practices do not seem to be “overcalling” the urgency of the nature of the problem when making referrals to specialists, or do not use the option of marking the urgency of a referral.

Specialists and family physicians in this study highlighted that wait time reporting would enable primary care physicians to more comprehensively consider the factors when creating a referral. Such factors may include: (1) having the shortest wait time is most important to reduce morbidity experienced by patients while waiting; (2) that geography is more important to a patient and some may prefer a longer wait time if it meant they could see a specialist closer to where they live; or (3) it may be that seeing a specific specialist is most important, and a patient would be willing to wait longer for their consultation to see a particular specialist. Wait time data may also be useful for physicians newer to practice or those without a preferred specialist in mind to learn about specialists in the region. Additionally, combining referral data across practices/regions could lead to better local, regional and provincial specialist access information for human resource planning purposes. This could complement other initiatives that may contribute to shortening specialist wait times including telehealth consults [22] (electronic asynchronous consultations obviating the need for face to face appointments between patient and specialist) and e-referral systems (i.e. province wide electronic health referral system where wait times could be viewed and referral status could be tracked), pre-assessment in specialized clinics [23] (a model of triage and appointment allocation to reduce wait times), and central intake [24] (instead of having multiple-queues and multiple-servers to manage referrals, specific specialists in a given jurisdiction would have a single queue allowing each patient to see the first available specialist) [25, 26]. In 2008, the Edmonton North Primary Care Network (PCN) developed a provincial e-referral system, which includes a specialist database with information on specialist referral requirements, forms and protocols, and tracks wait times [27]. A trend analysis of the referral wait time (defined as the time from referral by a family physician to an appointment date with a specialist) from 2009 to 2011 using the program database (*n*= 33,281 referrals) for 22 specialties showed a decrease in the overall wait time year over year [25]. The province of Ontario is currently introducing eReferral which is an electronic platform, integrated within a growing number of electronic health management systems but also available as a web-based platform, which allows secure referrals to be sent and received between family physicians and specialists [28]. eReferral enhances communication between primary care providers and specialists, eliminates the need for fax-based methods of correspondence, and displays specialist wait time information [29].

The United Kingdom’s National Health Service (NHS) tracks wait times from the time a patient is referred to a specialist by the family doctor to the time the patient receives medical treatment from a specialist. The NHS has set a benchmark: 90–95% of patients should wait no longer than 18 weeks from the time at which they are referred to the time when they are treated [11]. In comparing our median wait time data with the NHS 18 week benchmarks it was interesting to note that about 90% of patients had a wait time within the 18 week target. Applying benchmarks could be another way to shift the needle on wait times. A first step is to be able to have access to the wait time information, which is presently not easily extractable and publicly available by most EMR vendors.

This study demonstrated that wait time information, valued by family physicians and specialists, lives within electronic health records, but is not easily extractable without manual manipulation. Aside from time required to create the wait time reports manually, limitations of the current study include a high degree of missing data such as postal codes and urgency of the referral, which were used to derive income quintiles, material deprivation indices, and urgency of referral. The degree of missing data highlights the need to have these fields entered in a codifiable format in the EMR. The study also employed a small sample size (both in terms of unique patients and numbers of referrals) and only a single EMR. The same level of detailed referral data may not be available in all EMRs. The study setting focused on the Greater Toronto Area, and is likely not representative of wait times for a broader geography. The study also did not consider the time to make the referral in the first place (wait time 0), before it was sent to the specialist and the appointment was booked. However, the primary objective of the study was not to analyze the wait times themselves, but to explore the feasibility of extracting wait time data from the EMR and the clinical utility of the information for family physicians and specialists. The heterogeneity with which referral information is captured in primary care EMRs is a hurdle to scaling up wait time reporting to provincial/national levels. However, this study highlights important potential uses of wait time information for providers, patients and health regulators, and next steps would include the development and expansion of systems, such as eReferral, to more accurately collect and report wait time data. Finally, the descriptive qualitative aspect of this research never intended to reach saturation of all ideas related to wait time reporting, but rather act as a starting point to obtain information about how family physicians and specialists’ view and value wait time information.

## Conclusion

Wait time information is perceived as valuable information to family physicians and specialists. While there are challenges with scaling up the functionality of various EMRs to provide wait time data, this study demonstrates that having access to specialist wait time information could aid provider and patient decision-making regarding specialist referrals, and potentially help with human resource planning and reduction of system bottlenecks. The use of technologies such as machine learning to code existing EMR data, or eReferral systems that readily report wait times, may improve the efficiency of creating wait time reports or sharing wait time information. Future work can be directed towards expanding primary care to specialist wait time reporting functionality through referral modalities such as e-referral systems. This way, family physicians and patients can make a more informed choice when considering the multitude of factors that go into the decision of which specialist to refer to.

## Supplementary Information

**Additional file 1.**

**Additional file 2.**

**Additional file 3.**

## Data Availability

The datasets generated and/or analysed during the current study are not publicly available due to confidentiality but are available from the corresponding author on reasonable request.

## Abbreviations

EMR: Electronic medical record
CPSO: College of Physicians and Surgeons of Ontario
UTOPIAN: University of Toronto Practice-based Research Network
PCN: Primary Care Network
NHS: National Health Service
