# Supplementary material for: Specialist wait time reporting using family physicians’ electronic medical record data: a mixed method study of feasibility and clinical utility
Source: BMC Prim Care. 2022 Apr 7;23:72. doi: 10.1186/s12875-022-01679-x (PMC8988329; doi:10.1186/s12875-022-01679-x)
Supplement: Supplementary file 1 — Additional file 1. [file 12875_2022_1679_MOESM1_ESM.pdf]

Appendix 1: Sample Wait Time Report by Specialty

Site	Specialist	Department	Address	Specialist City	Number of Referrals	Median Wait Time (Days)
MSH	Dr. 1	Gastroenterology	123 Edward Street, Suite 825	Toronto	59	15
MSH	Dr. 2	Gastroenterology	123 Edward Street, Suite 825	Toronto	96	150
MSH	Dr. 3	Gastroenterology	123 Edward Street, Suite 825	Toronto	1	3
MSH	Dr. 4	Gastroenterology	123 Edward Street, Suite 825	Toronto	4	3
MSH	Dr. 5	Gastroenterology	123 Edward Street, Suite 825	Toronto	3	5
MSH	Dr. 6	Gastroenterology	123 Edward Street, Suite 825	Toronto	3	7
MSH	Dr. 7	Gastroenterology	123 Edward Street, Suite 825	Toronto	1	9
MSH	Dr. 8	Gastroenterology	123 Edward Street, Suite 825	Toronto	12	20.5
MSH	Dr. 9	Gastroenterology	123 Edward Street, Suite 825	Toronto	12	57
MSH	Dr. 10	Gastroenterology	123 Edward Street, unit 400	Toronto	24	63
MSH	Dr. 11	Gastroenterology	1366 Yonge St #301	Toronto	43	135
MSH	Dr. 12	Gastroenterology	1366 Yonge St #301	Toronto	3	6
MSH	Dr. 13	Gastroenterology	1366 Yonge St #301	Toronto	1	27
MSH	Dr. 14	Gastroenterology	1366 Yonge St #301	Toronto	4	27.5
MSH	Dr. 15	Gastroenterology	1366 Yonge St #301	Toronto	1	28
MSH	Dr. 16	Gastroenterology	1366 Yonge St #301	Toronto	1	28
MSH	Dr. 17	Gastroenterology	1366 Yonge St #301	Toronto	6	29
MSH	Dr. 18	Gastroenterology	1366 Yonge St #301	Toronto	4	30
MSH	Dr. 19	Gastroenterology	1366 Yonge St #301	Toronto	9	34
MSH	Dr. 20	Gastroenterology	1366 Yonge St #301	Toronto	4	37
MSH	Dr. 21	Gastroenterology	1366 Yonge St #301	Toronto	73	66
MSH	Dr. 22	Gastroenterology	1366 Yonge St #301	Toronto	4	120
MSH	Dr. 23	Gastroenterology	1366 Yonge St, Suite 301	Toronto	1	78
MSH	Dr. 24	Gastroenterology	1600 Steeles Ave. W.	Vaughan	163	28
MSH	Dr. 25	Gastroenterology	1600 Steeles Avenue West, Unit 35	Concord	1	9
MSH	Dr. 26	Gastroenterology	1600 Steeles Avenue West, Unit 35	Concord	1	14
MSH	Dr. 27	Gastroenterology	2038 VICTORIA PARK AVENUE	TORONTO	4	10
MSH	Dr. 28	Gastroenterology	3030 Lawrence Ave E	Scarborough	1	12
MSH	Dr. 29	Gastroenterology	330 HWY 7 EAST SUITE 510	RICHMOND HILL	1	3
MSH	Dr. 30	Gastroenterology	4800 Leslie Street, Suite 401	North York	8	51

Lunenfeld-Tanenbaum Research Institute
Sinai Health System

Appendix 1:Sample Wait Time Report
Median Wait Times by Specialty

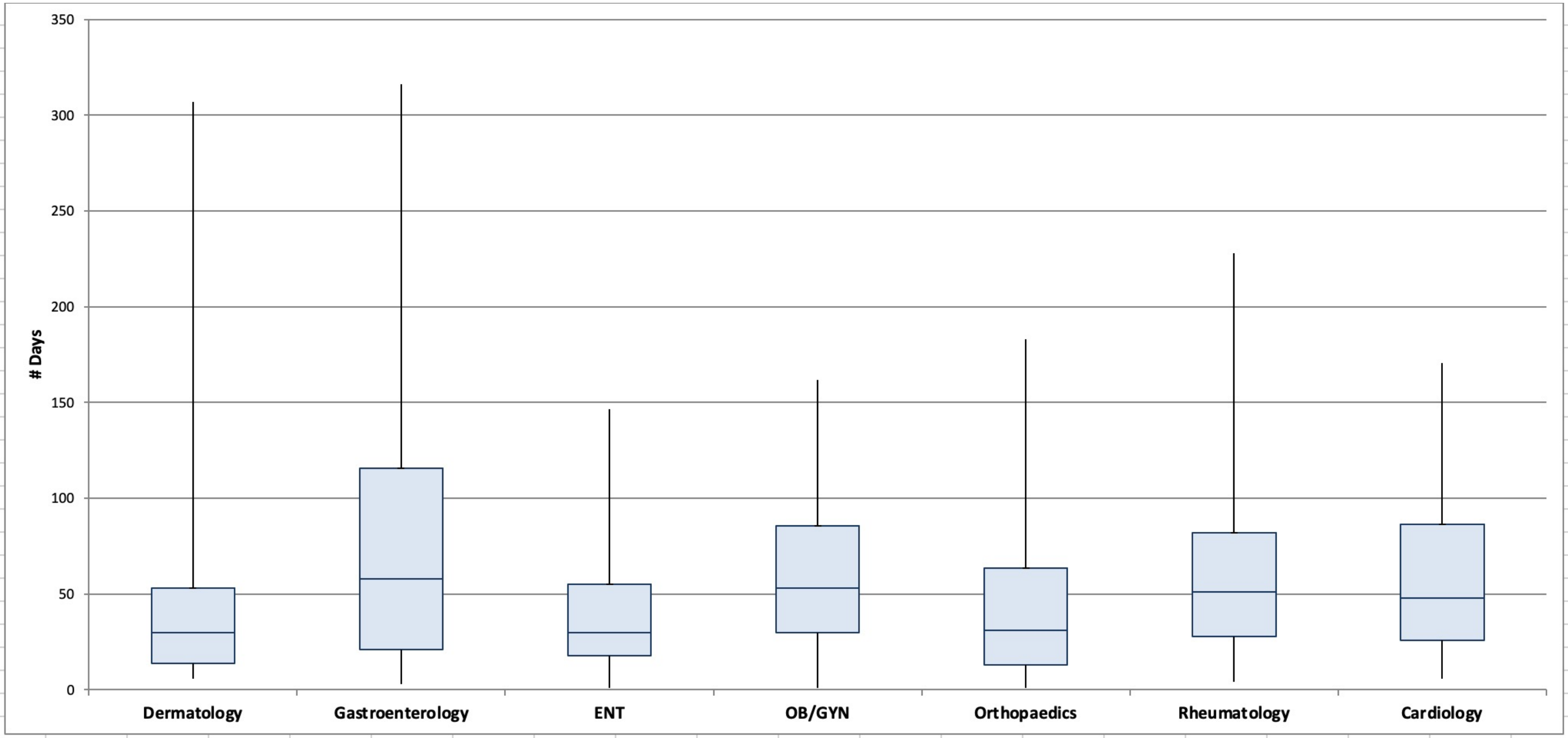

	Dermatology	Gastroenterology	ENT	OB/GYN	Orthopaedics	Rheumatology	Cardiology
Range	6.00 - 308.00	3.00 - 358.00	1.00 - 146.50	1.00 - 131.00	1.00 - 183.00	4.00 - 228.00	6.00 - 170.50
Quartiles:							
25%	15.00	21.00	18.00	29.75	13.00	28.00	26.00
50%	31.00	55.00	30.00	52.00	31.00	51.00	48.00
75%	54.00	112.50	55.00	84.75	63.50	82.00	86.50

Lunenfeld-Tanenbaum Research Institute
Sinai Health System
