# Supplementary material for: Specialist wait time reporting using family physicians’ electronic medical record data: a mixed method study of feasibility and clinical utility
Source: BMC Prim Care. 2022 Apr 7;23:72. doi: 10.1186/s12875-022-01679-x (PMC8988329; doi:10.1186/s12875-022-01679-x)
Supplement: Supplementary file 2 — Additional file 2. [file 12875_2022_1679_MOESM2_ESM.pdf]

Appendix File 2: **Semi-structured Interview Guide for Family Physician Focus Groups**

Theme: Relevance

1. What do you think of this information?
   Probe: What aspects do you find important, not important or confusing?
   Is there anything missing - would you like to see information that is not in this report?

2. How interested are you in knowing wait times from primary care to specialist care in your community?
   Probe: Would you like to receive this data for your practice?
   How often would you be interested in seeing your wait time data?

3. What relevance if any does the information in this report have in your practice?
   Probe: What is particularly useful?
   Would you change your referral patterns based on this information?
   How else could this information have relevance in your practice?

4. In general, how important is this information for our healthcare system?

Theme: Clinical Utility

5. Is there anything you feel is not useful to you in this report?
6. Are there any barriers that would prevent you from using this information in your practice?

7. Would you find it useful to learn about new specialists from this report?
8. Do you think that benchmarks should be set for wait times for urgent, semi-urgent and non-urgent referrals from primary care to specialist care?
   Probe: What do you think a reasonable wait time should be for each category?

9. Do you think there should be public reporting of wait times (from primary care referral to specialist appointment)?
   Probe: If yes or no, why?

Theme: Acceptability of the report

10. What are your impressions on how this report is presented?
    Probe: Is this report clear?
    How could we improve presentation of this information?

11. What would be your preferred method of receiving this information?
    Probe: Paper report in mail?
    Emailed?
    Available on-line?
    Available through an organization like Health Quality Ontario?

12. Do you see any potential harms in having this information available to you?

13. How likely are you to discuss this information with others if available to you?

14. Can you make suggestions for improvement?

Is there anything else you can think of regarding your perspective on this report?
