# Supplementary material for: Specialist wait time reporting using family physicians’ electronic medical record data: a mixed method study of feasibility and clinical utility
Source: BMC Prim Care. 2022 Apr 7;23:72. doi: 10.1186/s12875-022-01679-x (PMC8988329; doi:10.1186/s12875-022-01679-x)
Supplement: Supplementary file 3 — Additional file 3. [file 12875_2022_1679_MOESM3_ESM.pdf]

Appendix 3: **Semi-structured Interview Guide for Specialist Focus Groups**

Theme: Relevance

1. What do you think of this information?
   Probe: What aspects do you find important, not important or confusing?
   Is there anything missing - is there data you would like to see that is not included in the report?

2. How interested are you in knowing the wait times from primary care to specialist care in your community?
   Probe: Would you like to receive this data for your practice?
   How often would you want to see your wait time data?

3. How interested are you in learning about the wait times of your colleagues?

4. In general, how important is this information for our healthcare system?

Theme: Clinical Utility

5. What do you perceive to be clinical benefits of this information, if any?

6. Would this information change your practice in any way?
   Probe: Do you think this information would change how you book patients?
   How else may this information impact your work?

7. Would you do things differently if your wait time was much longer compared to your peers?
   Probe: Please explain

8. Are there any barriers that would prevent you from using this information in your practice?

9. Do you think that benchmarks should be set for wait times for urgent, semi-urgent and non-urgent referrals from primary care to specialist care?
   Probe: What do you think a reasonable wait times should be for each category?

Theme: Acceptability

10. What are your impressions on how this report is presented?
    Probe: Is this report clear?
    How could we improve the presentation of this information?

11. What would be your preferred method of receiving this information?
    Probe: Confidentially in a private meeting?
    Paper report in mail?
    Emailed?

Available on-line?
Through a meeting with your Chief?
Available through an organization like Health Quality Ontario?

12. Would you prefer individual identification of your wait times and confidential reporting for comparators? Or identification of your wait times and nominal reporting of comparators?

13. Do you see any potential harms in having this information available to you?

14. How likely are you to discuss this information with others if available to you?
    Probe: Your specialist colleagues? FDs? Chief?

15. Would you feel comfortable with public reporting of your wait times (from primary care referral to specialist appointment)?
    Probe: Please explain

16. Can you make suggestions for improvement?

Is there anything else you can think of regarding your perspective about this report?
